# Draft *De Novo* Genome Assembly of Acinetobacter pittii Strain VKPM B-3780, a Prospective Multifunctional Bioremediation Agent

**DOI:** 10.1128/MRA.00554-21

**Published:** 2021-08-12

**Authors:** A. O. Izotova, K. O. Petrova, A. A. Korzhenkov, A. A. Bavtushnyi, K. V. Sidoruk, E. V. Patrusheva, M. V. Patrushev, D. A. Kwon, S. V. Toshchakov

**Affiliations:** a National Research Center Kurchatov Institute, Moscow, Russia; b National Research Center Kurchatov Institute-GOSNIIGENETIKA, Moscow, Russia; University of Southern California

## Abstract

Acinetobacter pittii strain VKPM B-3780 is a prospective degrader of oil and methanol, isolated from industrial wastewater. Here, we present the draft genome sequence of strain VKPM B-3780, obtained using Illumina sequencing of the fragment genomic library.

## ANNOUNCEMENT

Acinetobacter pittii strain VKPM B-3780 was isolated in 1984 from industrial wastewater from a phenol-formaldehyde resin manufacturing plant and deposited in the Russian National Collection of Industrial Microorganisms (VKPM). Since then, several reports have been published noting its potential as a prospective oil degrader (1, 2). Furthermore, a microbial consortium comprising strain VKPM B-3780 was patented recently as a method for the biodegradation of methanol (3). Thus, due to the multipurpose biodegradation potential of this strain, its genome sequence might be valuable for further improvement to existing bioremediation technologies using targeted genetic engineering. Here, we present a draft genomic sequence of strain VKPM B-3780, obtained by Illumina sequencing.

For DNA isolation, strain VKPM B-3780 was cultivated aerobically at 30°C using nutrient broth medium. Genomic DNA was extracted as described previously (4) and further repurified using a PCR purification kit (Qiagen, Germany). Then, 0.5 ng of DNA was used for fragment library preparation using a Nextera XT DNA library prep kit (Illumina, San Diego, CA, USA) according to the manufacturer’s instructions. The library was sequenced on the MiSeq system using 2 × 250-bp paired-end chemistry. A total of 911,556 read pairs were obtained.

Default parameters were used for all software packages unless otherwise specified. Trimming of adapters and low-quality read regions was performed using CLC Genomics Workbench v.20.0.4 (Qiagen, Germany) with the Trim Reads tool with 0.01 maximum error probability and zero allowed ambiguous bases. As a result, 894,024 paired reads were used for the assembly. The reads were assembled using the SPAdes v.3.15.2 assembler in -isolate mode (5). The final assembly consisted of 96 contigs with a total length of 3,989,796 bp. The *N*_50_ metric of the assembly was 106,417 bp. The final average coverage of the assembly was 88×. The GC content was 38.8%. Analysis of the assembly quality using CheckM (6) predicted 100% completeness and 0% contamination.

Although strain VKPM B-3780 was initially classified as Acinetobacter calcoaceticus, genome-based taxonomy placement using the GTDB-Tk package (7) classified this strain as Acinetobacter pittii (8) with an average nucleotide identity (ANI) value with the Genome Taxonomy Database (GTDB) species representative genome of 96.92% and an aligned fraction of 90%. Assembly annotation was performed using the Prokaryotic Genome Annotation Pipeline during submission to the NCBI database (9). In total, 3,894 genes were predicted, including 3,735 protein-coding genes, 82 pseudogenes, and 77 RNA genes.

Analysis of the genes that might be responsible for the biodegradation of oil and other pollutants was performed using the NCBI-provided annotation and functional annotation made using the RAST Web server (10). The analysis revealed the presence of at least one *benABC* operon (VKPMB3780_12375 to VKPMB3780_12400) initiating benzoate catabolism, an *antABC* operon (VKPMB3780_15480 to VKPMB3780_15490) encoding anthranilate dioxygenase complex, and one multicomponent phenol-hydroxylase coding gene cluster (VKPMB3780_12415 to VKPMB3780_12450). The metabolism of alkanes might be initiated by the action of alkane-1-monooxygenase (VKPMB3780_06570).

Taken together, these preliminary results highlight the potential of strain VKPM B-3780 as a versatile bioremediation agent and offer key insights into the genetic factors determining its metabolism.

### Data availability.

The data are publicly available at NCBI GenBank. The BioSample, BioProject, and assembly whole-genome sequencing (WGS) accession numbers are SAMN19313528, PRJNA732173, and JAHEOK000000000.1, respectively. The raw sequencing reads are available in the NCBI SRA under accession number SRR14655868.
